# The -144C/A Polymorphism in the Promoter of HSP90beta Is Associated with Multiple Organ Dysfunction Scores

**DOI:** 10.1371/journal.pone.0058646

**Published:** 2013-03-13

**Authors:** Yan Zhao, Liju Tao, Dongpo Jiang, Xingyun Chen, Ping Li, Yalei Ning, Renping Xiong, Ping Liu, Yizhi Peng, Yuan-Guo Zhou

**Affiliations:** 1 The Molecular Biology Center, State Key Laboratory of Trauma, Burn and Combined Injury, Research Institute of Surgery and Daping Hospital, Third Military Medical University, Chongqing, People's Republic of China; 2 State Key Laboratory of Trauma, Burn and Combined Injury, Institute of Burn Research, Southwest Hospital, the Third Military Medical University, Chongqing, People's Republic of China; 3 Intensive Care Unit, Daping Hospital, Third Military Medical University, Chongqing, People's Republic of China; Harvard Medical School, United States of America

## Abstract

**Introduction:**

Variations in genetic background are the leading cause of differential susceptibility to traumatic infection. Heat shock protein 90 (HSP90), a broadly distributed and conserved molecule, regulates inflammation under stressful and traumatic conditions. However, the relationships between HSP90 genetic polymorphisms, post-traumatic inflammatory responses and organ function remain unknown.

**Methods:**

A total of 286 healthy volunteers and patients with severe trauma took part in a single nucleotide polymorphism (SNP)-based analysis of the HSP90beta gene and a clinical association analysis. HSP90beta and TNF-alpha levels were determined using quantitative PCR and western blot. The transcriptional activity of the HSP90beta promoter was assayed using the Dual-Luciferase Reporter Assay System.

**Results:**

The minor allele frequencies for the SNP located at −144 bp relative to the HSP90beta transcriptional start site were 28.47% and 28.52% in the normal and trauma populations, respectively; no significant differences were found between these two distributions. However, the results showed that a promoter containing the -144A allele had a higher transcriptional activity than did a promoter containing the wild-type -144C allele. Furthermore, the -144A promoter induced high expression of HSP90beta and low expression of the inflammatory factor TNF-alpha in a lipopolysaccharide-induced inflammatory model. A clinical association analysis showed that the multiple organ dysfunction scores for -144AA genotype carriers were significantly lower than those of -144CC carriers following trauma. No significant correlations were found between the presence of the two alleles and the incidence of sepsis.

**Conclusions:**

These results indicate that differences in expression caused by the -144 polymorphism in the HSP90beta promoter are associated with cellular inflammatory responses and the severity of organ injury. These findings will aid in risk assessment and early prevention of complications for patients with severe trauma.

## Introduction

High mortality caused by severe trauma and secondary consequences, such as sepsis and multiple organ dysfunction syndrome (MODS), is a difficult problem in clinical practice. A growing number of scientists believe that in addition to more traditional treatment methods future therapeutic strategies will be directed toward individual differences in patient's genetic backgrounds. To date, many studies have reported that genetic polymorphisms in a variety of pattern recognition receptors [1]–[3], cytokines [4]–[6] and coagulation-related molecules were found to be associated with degree of inflammation and patient prognosis [7]–[9]. Recent studies have indicated that HSP90, a widely expressed stress protein, plays an important regulatory role in a number of inflammatory signaling pathways. For example, HSP90 regulates the expression of inflammatory factor TNF-alpha, IFN-gamma, and IL-1 (via NF-kappaB), and it also modulates the SAPK/JNK signaling pathway in lipopolysaccharide (LPS)-induced inflammatory mouse models [10], [11]. HSP90 also affects the cytoskeletal protein actin and enhances disease development in acute lung injury models [12], [13]. Therefore, mutations in the HSP90 gene may play a more significant role than modulators involved in single inflammatory signaling pathways. However, because the HSP90 protein is highly conserved and shows few differences between species, little research has been carried out regarding the effects of HSP90 mutations.

Existing data on HSP90 mutations are mainly limited to observations in yeast [14]–[18], and human HSP90 gene mutations have only been reported for Caucasian populations [19]. Human HSP90 has two isoforms in the cytoplasm: the constitutively expressed HSP90beta and the inducible HSP90alpha (equivalent to mouse HSP84 and HSP86, respectively) [20], [21]. Although these two isoforms share certain common functions, they have distinct characteristics: HSP90beta is primarily involved in signal transduction, growth and development [22], whereas HSP90alpha plays a role in the heat-shock response [14]. In a previous study, we showed that BALB/c and C57BL/6 mice had different tolerances for trauma and that their HSP84 genes differed at several functional sites [23]; eliminating these genetic changes lessened or eliminated the differences in trauma tolerance between the two mouse strains, and we also showed that these changes affected the activity of glucocorticoid receptor (GR), a client protein of HSP90 [23]. In addition, experiments have shown that mice transfected with a plasmid expressing HSP84 have a higher stress tolerance and a significantly lower injury rate; furthermore, the degree of GR nuclear translocation was associated with HSP84 genotype [24]. These results indicate that the observed differences in damage between the two mouse strains were affected by changes in the HSP84 gene. Therefore, despite the fact that HSP90beta is highly conserved, we hypothesized that polymorphisms of this gene may still exist in the human population. As a strongly expressed intracellular protein, these putative variations may have noticeable effects on the inflammatory response and organ function following trauma.

In this study, we examined SNPs in the promoter of the HSP90beta gene in healthy Han populations as well as in severe trauma patients living in Chongqing, China. We confirmed that these SNPs affected the transcription of HSP90beta and that expression of this gene impacts the inflammatory response. Finally, we correlated the presence of specific SNPs with the degree of injury in patients with severe trauma to further clarify the role of HSP90beta SNPs in the inflammatory response following trauma and MODS.

## Materials and Methods

### Study populations

The study populations consisted of 144 healthy blood donors and 142 major trauma patients admitted to the clinical laboratory and ICU of Daping Hospital and the Burn Department of Southwest Hospital. The healthy population was composed of 96 men and 48 women with a median age of 37.8 yrs (range 23–78 yrs). The trauma patient population was composed of 107 men and 35 women with a median age of 42.1 yrs (range 19–84 yrs). All participants were Han Chinese living in Chongqing, and the trauma patients were chosen to meet the following criteria: an Injury Severity Score (ISS) ≥16; aged 18–85 yrs; a survival time >1 week; no previous blood transfusions; and no pre-existing respiratory, cardiovascular, hepatic, renal, hematological or immunological conditions. ISSs were calculated according to the Abbreviated Injury Scale, 1988 Revision [25], and MOD (multiple organ dysfunction) scores were evaluated based on Marshall's method [26].

### Ethics statement

The study protocol was reviewed and approved by the Ethical and Protocol Review Committee of Third Military Medical University, and written informed consent was obtained from each patient or his or her next of kin.

### Genotyping

Whole blood was collected using EDTA-K2 anticoagulation, and genomic DNA was extracted using the phenol/chloroform method. The sequence of the HSP90beta promoter was obtained from the Eukaryotic Promoters Database (http://www.epd.isb-sib.ch/). The primers used to amplify the HSP90beta promoter (−1102 to 210 bp) were based on the sequence AMYH01016321 from the NCBI database and were as follows: 5′-GAGCTCCGGCTGCCCTGCACT-3′ (Forward) and 5′-CACAAGCCACCCCGACATCT-3′ (Reverse). PCR amplification was performed using the following conditions: 94°C for 5 min, followed by 35 cycles of 94°C for 30 s, 58°C for 30 s, and 72°C for 1.5 min, with a final extension time of 72°C for 10 min; the final product was 1312 bp. To ensure accuracy, each sample was sequenced twice (Shanghai Genesky Bio-Tech Co.), and genotyping was performed by researchers unfamiliar with the nature of the experiments.

### Transcriptional activity

Two HSP90beta promoter fragments were produced using PCR amplification of genomic DNA from two individuals homozygous for either the -144CC or -144AA HSP90 alleles. The primers and PCR conditions are described above. These products were cloned into the pMD19-T vector. The resulting plasmids were cleaved with the restriction endonucleases *HindIII* and *SacI*, and the promoter fragments were sub-cloned into the pGL3-Basic vector (Promega, USA) a promoter-free plasmid containing the firefly luciferase reporter gene. These vectors were transiently transfected into cultured U937 cells using the Lipofectamine 2000 kit (Invitrogen, USA), and transcriptional activities of the HSP90beta promoters were determined using the Dual-Luciferase Reporter Assay System (Promega, USA) 24 h after transfection with or without a heat-stress at 42°C for 1 h. The pRL-CMV vector containing a renilla luciferase reporter gene was co-transfected with the pGL3 vectors for normalization purposes; an empty pGL3 vector (pGL3-Basic) and a pGL3 vector containing the SV40 promoter (pGL3-SV40) were used as negative and positive controls, respectively. Transcriptional activity was measured using a Luminoskan Ascent luminometer (Thermo Labsystems, Finland) and measurements are reported as the ratio of the firefly and renilla luciferase activities.

### Ex vivo stimulation with LPS

Three milliliters of whole blood was collected from each healthy individual, 1 ml of which was anticoagulated with EDTA-K2 and used for DNA extraction and HSP90beta genotyping. The remaining 2 ml of blood was anticoagulated with 100 U of heparin and mixed with an equal volume of RPMI-1640 medium containing 10% FCS at 37°C. The cells were divided into LPS (treated with 200 ng/ml LPS) and placebo (mixed with an equal volume of saline) groups; cells were assayed 3 h after LPS stimulation.

### Quantitative RT-PCR

Quantitative RT-PCR was used to determine the mRNA levels of TNF-alpha and HSP90beta. Total RNA was extracted using the TRIzol RNA extraction kit (Invitrogen, USA) according to the manufacturer's instructions. The primers used to amplify TNF-alpha and HSP90beta were designed based on the NM_000594 and NM_007355 sequences from the NCBI database, respectively, and were as follows: 5′-CACGCTCTTCTGCCTGCT-3′ (Forward) and 5′-GCTTGTCACTCGGGGTTC-3′ (Reverse) for TNF-alpha; and 5′-AACTCTATGTCCGCCGTGTG-3′ (Forward) and 5′-CTTCTGCCAGCTCAGAGAAG-3′ (Reverse) for HSP90beta. The primers used to amplify GAPDH (NM_002046), a house-keeping gene, were 5′-GAAGGTGAAGGTCGGAGTC-3′ (Forward) and 5′-GAAGATGGTGATGGGATTTC-3′ (Reverse). The samples were denatured by heating at 95°C for 30 s, followed by 40 cycles of 95°C for 30 s, 58°C for 30 s and 72°C for 30 s. HSP90beta and TNF-alpha mRNA levels were normalized against the house-keeping gene GAPDH, and relative expression levels were determined by comparing expression to the wild-type (-144C) allele without LPS stimulation using the equation 2^−△△Ct^.

### Immunoblotting analysis

The samples were separated on 10% SDS-polyacrylamide gels and transferred to nitrocellulose membranes. The anti-HSP90beta and anti-beta-actin antibodies were purchased from Santa Cruz Biotechnology (sc-7947 and sc-8432, respectively). HRP-conjugated secondary antibodies for HSP90beta (A6154) and beta-actin (A4416) were purchased from Sigma, and the ECL Plus Western Blotting Detection System (RPN2132) was purchased from Amersham. Band density was measured on a UVP Bioimaging acquisition and image-analysis system (Ultra-Violet Products Ltd, UK) using beta-actin bands for normalization.

### Statistical analysis

Allele frequencies were calculated by gene counting. Genotype distribution was tested for deviation from Hardy-Weinberg (HW) equilibrium using the χ^2^ test. A one-way analysis of variance (ANOVA) was used to compare transcriptional activity, ISS and MOD score for TNF-alpha and HSP90beta in the various genotypes. We also tested for dominant (variant homozygotes+heterozygotes vs. wild homozygotes) and recessive (variant homozygotes vs. heterozygotes+wild homozygotes) effects using an ANOVA. Allele dose was defined as the number of copies of each variant allele present in the genotype. Linear regression analysis was performed to quantify the effect of allele dose; age and gender were used as covariates to exclude their influence. The association between genotype and the incidence of sepsis was analyzed using the χ^2^ test, and correlation analyses were performed using logistic regression analysis. All statistical analyses were performed using SPSS 11.0 software program. *P* values<0.05 were considered to be significant.

## Results

### Allele frequencies for the HSP90beta promoter in a Chinese Han population

**Figure 1:**
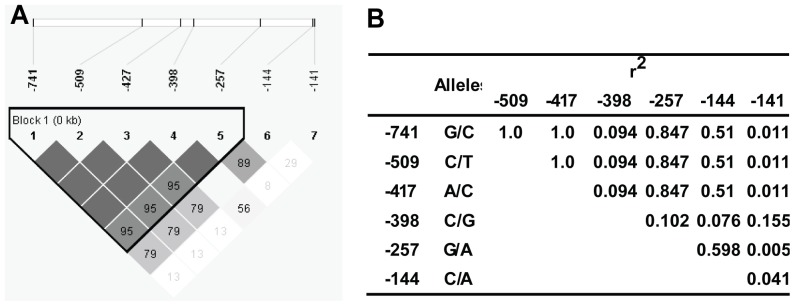
Overview of selected SNPs and their linkage disequilibrium (LD) statistics. (**A**) The locations of seven SNPs within the promoter of the HSP90beta gene. The data in the grids indicate D′ values. (**B**) Alleles of the seven SNPs and their r^2^ values. The results indicated that the five SNPs (−741, −509, −417, −398 and −257) were closely linked.

We identified the following seven high-frequency mutations in the tested samples: rs10948128, rs476632, rs3757283, rs1570920, rs9462978, rs9472238 and rs324131, which were located at −741, −509, −417, −398, −257, −144 and −141 bp relative to the HSP90beta gene (the transcriptional start site is +1 bp), respectively. The D′ values of the first 5 SNPs were greater than 0.95, indicating that they were closely linked with one another (Fig. 1). Therefore, rs10948128 (−741) was selected as the tag SNP and was examined in the following analyses. The genotype distributions of rs10948128 (−741), rs9472238 (−144) and rs324131 (−141) were determined for the healthy and trauma populations, and no significant differences were found between the two populations; the minor allele frequencies for the −144 site of the HSP90beta promoter in the normal and trauma populations were 28.47% and 28.52%, respectively (Table 1). The seven high-frequency HSP90beta SNPs identified in the Chongqing Han population were analyzed to determine whether they introduced or removed transcription-factor binding sites, and the results showed that the −144 SNP introduced a new high-affinity binding site for transcription factor early growth response protein 1 (EGR1), which would be predicted to promote transcription of HSP90beta. Therefore, we examined the expression of HSP90beta in subjects with different −144 HSP90beta genotypes.

**Table 1:** Distributions of 3 SNPs of the HSP90beta promoter in healthy and trauma patients.

	N	−741, N(%)			−144, N(%)			−141, N(%)		
		GG	GC	CC	CC	CA	AA	TT	TC	CC
Healthy	144	76(52.78)	61(42.36)	7(4.86)	73(50.69)	60(41.67)	11(7.64)	50(34.72)	63(43.75)	31(21.53)
				26.04^a^			28.47^a^			43.40^a^
Patients	142	82(57.75)	54(38.03)	6(4.25)	73(51.40)	57(40.14)	12(8.45)	52(36.62)	59(41.55)	31(21.83)
				23.24^a^			28.52^a^			42.61^a^
Total	286	158(55.24)	115(40.21)	13(4.55)	146(51.05)	117(40.91)	23(8.04)	102(35.66)	122(42.66)	62(21.68)
				24.65^a^			28.50^a^			43.01^a^

a indicates minor allele frequency (MAF).

### Expression of HSP90beta in peripheral blood leukocytes

**Figure 2:**
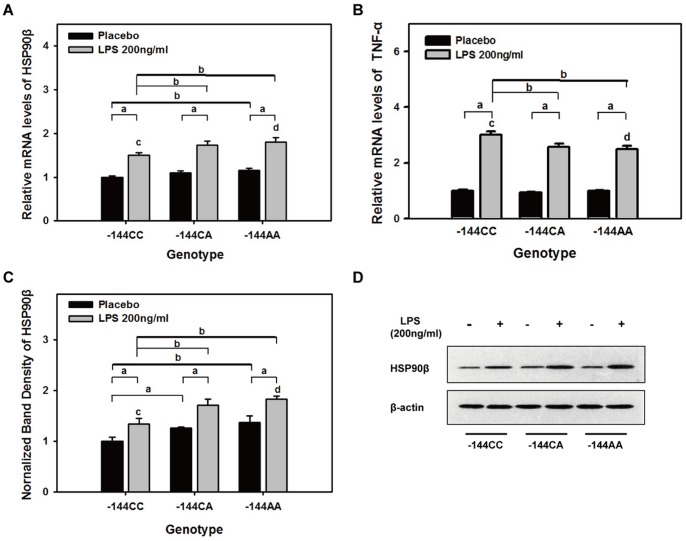
Relative levels of HSP90beta and TNF-alpha in the cultured white blood cells. Relative mRNA levels of HSP90beta (**A**) and TNF-alpha (**B**) in cultured white blood cells (WBC) from healthy patients were assayed using quantitative PCR. Relative mRNA levels were normalized to the house-keeping gene GAPDH and quantified relative to the wild-type (-144CC) levels without LPS stimulation. Normalized band densities of HSP90beta protein (**C**) and representative immunoblots from healthy-patient samples (**D**). Data were normalized to beta-actin and quantified relative to the wild-type (-144CC) levels without LPS stimulation. WBCs were incubated with 200 ng/ml LPS at 37°C for 3 h. *^a^p*<0.01 between the two groups; *^b^p*<0.05 between the two groups; *^c^p* = 0.017 for A, *p* = 0.033 for B and *p* = 0.001 for C, the dominant effect (variant homozygotes+heterozygotes vs. wild-type homozygotes) was analyzed using a one-way ANOVA; *^d^p* = 0.109 for A, *p* = 0.07 for B and *p* = 0.061 for C, the recessive effect (variant homozygotes vs. heterozygotes+wild-type homozygotes) was analyzed using a one-way ANOVA. The results are shown as the mean±SEM values from three independent experiments.

Quantitative PCR analyses showed that in peripheral blood leukocytes of healthy subjects without LPS stimulation (*p* = 0.031, <0.05), HSP90beta mRNA levels in -144-variant homozygote (-144AA) carriers was significantly higher than in wild-type (-144CC) and variant heterozygote (-144CA) carriers (Fig. 2A). There was a significant difference between the variant homozygote and wild-type genotypes, which demonstrated that the variant had a higher basal level of HSP90beta transcription under normal conditions.

Three hours after the administration of LPS, HSP90beta mRNA levels were significantly higher in the LPS group compared with the placebo group (*p*<0.01), and furthermore, mRNA levels were lower in the wild-type group than in the homozygotes (*p* = 0.042, <0.05) and variant heterozygotes (*p* = 0.024, <0.05). These data demonstrated that individuals carrying mutations at the −144 site had increased expression of HSP90beta, which helps resist excessive damage due to severe inflammatory stress. The linear regression test showed a linear correlation between genotype and HSP90beta expression following LPS stimulation (F = 6.34; *p* = 0.02, <0.05). Furthermore, we found evidence for dominant effects using the linear model (F = 6.734; *p* = 0.017, <0.05) but recessive effects were not observed (F = 2.784; *p* = 0.109, >0.05).

Western blotting analyses were consistent with the quantitative PCR results, and we found a linear correlation between genotype and HSP90beta protein levels following LPS stimulation (F = 27.028; *p* = 0.001, <0.01). Furthermore, we found evidence for dominant effects using the linear model (F = 29.827; *p* = 0.001, <0.01) but recessive effects were not observed (F = 4.952; *p* = 0.061, >0.05).

### Expression of TNF-alpha in peripheral blood leukocytes

To demonstrate that changes in HSP90beta expression affected the degree of the inflammatory reaction, we measured TNF-alpha mRNA levels in peripheral blood leukocytes 3 h after LPS stimulation. TNF-alpha mRNA levels were significantly higher in the wild-type group than in homozygotes (*p* = 0.011, <0.05) and heterozygotes (*p* = 0.03, <0.05) (Fig. 2B), showing that the −144 SNP played a cytoprotective role by decreasing levels of TNF-alpha. Using linear regression, we showed a negative linear relationship between TNF-alpha mRNA levels and HSP90beta genotype (F = 5.883; *p* = 0.024, <0.05). In addition, increased TNF-alpha mRNA levels showed a dominant effect (F = 5.184; *p* = 0.033, <0.05) rather than a recessive effect (F = 3.625; *p* = 0.07, >0.05).

### The effects of the −144 SNP on HSP90beta transcription

**Figure 3:**
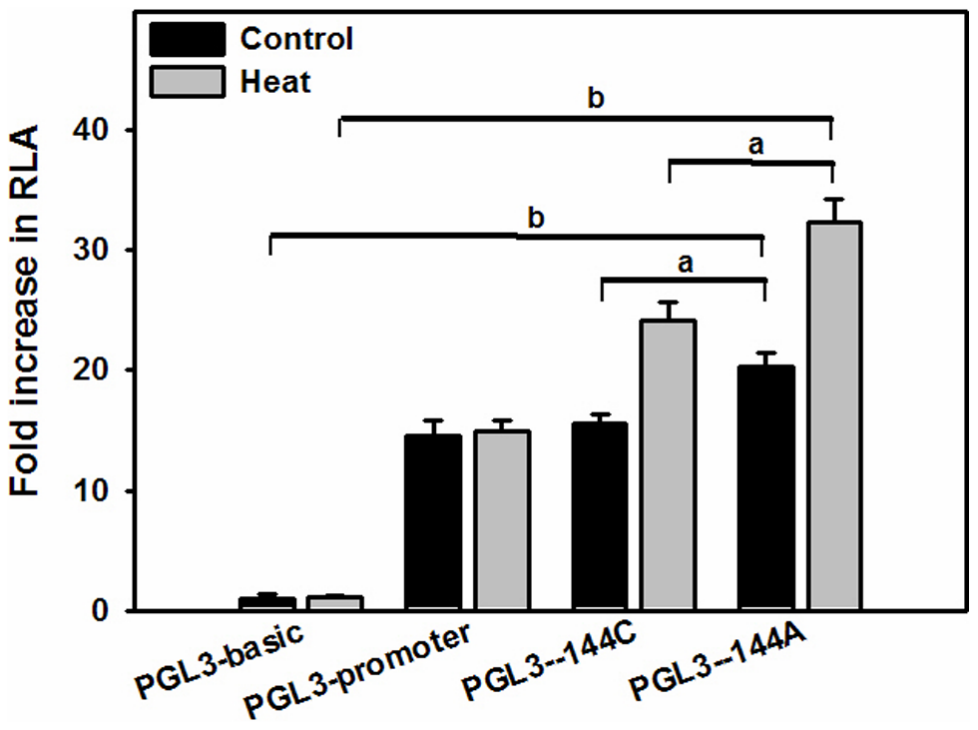
Effects of the −144 SNP of the HSP90beta promoter on transcriptional activity. Luciferase activity was normalized to control plasmid (pRL-CMV) expression. The results are shown as the mean±SEM (n = 4) values. The HSP90beta promoter vectors pGL3-144C (major allele) and pGL3-144A (minor allele) were transfected into U937 cells. pGL3-Basic and pGL3-SV40 were used as negative and positive control vectors, respectively. Relative luciferase activity was measured 24 h post-transfection with or without heat stress at 42°C for 1 h. Data are expressed as the fold-increase in luciferase activity relative to pGL3-Basic. A one-way ANOVA was used to assess statistical significance. *^a^p*<0.05, *^b^p*<0.01 between the two groups.

To further elucidate the effects of the −144 SNP on HSP90beta transcription, we analyzed the transcriptional activity of its promoter. We found that U937 cells transfected with a pGL3-HSP90beta plasmid containing a promoter with the −144 SNP had higher levels of luciferase activity than cells transfected with either the wild-type pGL3-HSP90beta promoter, negative control (no promoter) or positive control (SV40 promoter) plasmids, under both heat-stress (42°C) and normal conditions (*p*<0.05; Fig. 3). Therefore, we conclude that increased HSP90beta transcription mediated the protective effects of the −144 SNP. Next, we performed a clinical correlation analysis using a cohort of trauma patients.

### Clinical correlation analysis

Based on the inclusion criteria, we enrolled a total of 142 patients (mean age = 42.1±14.5 yrs) who had suffered severe trauma (mean ISS = 29.6±15.2); their clinical characteristics are shown in Table 2. In the studied population, 72 subjects had ISS values ranging from 16–25, and 70 subjects had an ISS value greater than 25. Among these individuals, 51 patients (35.9%) developed sepsis, and 8 patients (5.6%) died in the ICU. Statistical analyses indicated that MOD score was negatively correlated with the presence of the −144 SNP, which showed an allele-dose effect when the data were adjusted for sex, age and ISS (y = 2.957−0.9x; *p* = 0.010). Analysis of the MOD score using the dominant and recessive effect models indicated that the −144 SNP showed a dominant effect (*p* = 0.011, <0.05) rather than a recessive effect (*p* = 0.17, >0.05). In addition, we did not observe a significant relationship between genotypes carrying the −741 and −141 SNPs and MOD score (*p*>0.05). Moreover, tests showed no significant relationship between the −741, −144 and −141 SNPs and the incidence of sepsis (Table 3). Therefore, only the −144 SNP had an obvious effect on trauma patient phenotype, with mutation carriers showing a lower extent of injury than wild-type carriers.

**Table 2:** Clinical characteristics of patients with severe trauma (n = 142).

Age [avg. yrs (range)]	42.1±14.5(19–84)
Male/Female	107/35
ISS	29.6±15.2
16≤ISS<25 (n)	72
≥25 (n)	70
MODS (n, %)	40 (28.2)
Sepsis (n, %)	51 (35.9)
Non-survive (n, %)	8 (5.6)

ISS, Injury Severity Score; MOD, multiple organ dysfunction.

**Table 3:** Clinical relevance of 3 SNPs of the HSP90beta promoter in severe trauma patients.

Sites	genotype	Gender (F/M)	Age(yr)	ISS	MOD Score	Sepsis n(%)	HWE *p* value
−741	GG	14/68	42.3±15.1	30.6±15.0	2.26±0.39	29(35.4)	
	GC	19/35	41.6±14.0	28.6±14.4	1.63±0.40	20(37.0)	0.432
	CC	1/6	46.0±10.4	24.5±10.6	2.10±0.40	2(33.3)	
−144	CC	13/60	42.3±15.2	31.7±16.2	2.26±0.43	26(35.6)	
	CA	19/38	42.7±13.8	28.5±12.7	1.61±0.37	21(36.8)	0.853
	AA	3/9	38.0±12.8	22.2±6.9	1.58±1.06	4(33.3)	
					^abc^		
−141	TT	11/41	41.9±14.9	30.8±15.1	2.55±0.53	18(34.6)	
	TC	15/44	41.2±13.2	29.7±15.1	1.85±0.44	21(35.6)	0.073
	CC	8/23	44.1±16.3	27.4±12.6	1.13±0.37	12(38.7)	

a *p* = 0.011, the dominant effect (variant homozygotes + heterozygotes vs. wild-type homozygotes) was analyzed using a one-way ANOVA; *^b^p* = 0.17, the recessive effect (variant homozygotes vs. heterozygotes+wild-type homozygotes) was analyzed using a one-way ANOVA; *^c^p* = 0.01, the allele dose effect was analyzed using linear regression analysis; HWE, Hardy-Weinberg equilibrium; χ^2^ analysis was used to test for deviation from HWE; F, female; M, male; ISS, Injury Severity Score; MOD, multiple organ dysfunction.

## Discussion

We found that several SNPs were present in the promoter of the HSP90beta gene, despite the fact that this conserved molecular chaperone is constitutively expressed. Among these SNPs, some are found at high-frequency in the Chongqing population (e.g., rs10948128). This suggests that the conserved nature of HSP90 is primarily reflected in sequence and conformational conservation at the protein level; by examining HSP90 genetic sequences, and the promoter in particular, we find a number of mutations in our data as well as in the Genbank sequences. SNPs located within promoters, which are regions important for transcriptional regulation, can lead to changes in protein levels and, ultimately, function. No SNPs were observed in the core regions of the promoter, such the TATA-box or GC-box, although we did identify a SNP located at −144 bp relative to the HSP90beta start site (rs9472238) that created a new binding site for the transcription factor EGR1, likely leading to increased transcription of HSP90beta. Comparing our data with data from Genbank, we found that the occurrence of the −144 SNP in our subjects was similar to that of Asians in general, but this SNP was rarely observed in Africans, presumably due to ethnic differences.

We showed that the wild-type pGL3-144C vector had a similar transcriptional efficiency to that of the pGL3-SV40 vector but that it had lower activity than the pGL3-144A vector, which carried the variant promoter. These findings were consistent with the bioinformatics analyses, which predicted that the −144 SNP creates a binding site for EGR1 and would lead to increased transcription of the HSP90beta gene; EGR1 had not been previously reported to regulate expression of the HSP90beta gene. In addition, quantitative PCR analysis showed that individual carrying different −144 SNP genotypes differed markedly in their expression of the HSP90beta gene, both in the presence of absence of LPS administration, although differences were more obvious following LPS stimulation. For example, there was a significant difference between wild-type and variant genotypes including homozygotes and heterozygotes following LPS stimulation, but we only observed a difference between wild-type and homozygous-variant genotypes without LPS stimulation, which showed a dominant hereditary pattern. Therefore, we conclude that the −144 SNP affects expression of HSP90beta by altering its transcriptional activity, which ultimately affects the inflammatory reaction.

Cellular experiments and clinical correlation analyses were performed to verify the effects of the −144 SNP on HSP90beta function. Cellular experiments showed that different genotype carriers displayed had different degrees of inflammation, as measured by the expression of TNF-alpha following LPS stimulation. Furthermore, the analyses of clinical relevance were consistent with the cellular experiments. In other words, trauma patients carrying the wild-type -144C allele had higher MOD scores than patients carrying the variant -144A allele. However, we found no association between the incidence of sepsis and presence of the −144 SNP. As HSP90beta is only one factor in the complex mechanisms of sepsis, this may explain why the −144 SNP did not show a clinical correlation with sepsis in the current study.

Importantly, the −144 SNP in the HSP90beta promoter had an effect the inflammatory response and the severity of multiple organ dysfunction following trauma. HSP90beta exerts protective effects not only through its role as a chaperone for damaged proteins but also by regulating various signaling molecules related to inflammation. For instance, HSP90beta modulates the expression of TNF-alpha, IL-1beta, IL-6, mCD14 and NO by affecting NF-κappaB and its inhibitors, such as IkappaB kinase [11], P38 [27], NOS [28], [29] and JNK [30], [31]. Increased levels of heat shock proteins help to reduce damage in mononuclear cells [32], cardiac myocytes [33]–[35], coronary endothelial cells [36], skin [37], the gastrointestinal tract [38], nervous system [39] and embryonic cells [40], although the exact mechanisms of their action remain unclear. Glucocorticoid receptor (GR), an important client protein of HSP90, is a key effector molecule in the hypothalamic-pituitary-adrenal axis (HPA) and plays important roles in inflammatory reactions [41]–[43] and innate immunity [44]–[46]; therefore, the exact nature of the protective role of HSP90 in these processes remains unclear. We have speculated that the protective effects of HSP90 following traumatic infection were related to regulation of the GR pathway, which was consistent with our existing data that HSP90beta polymorphisms were associated with the nuclear translocation of GR and cellular tolerance to heat stress [23], [24], [47]. Functioning primarily as a chaperone, HSP90beta could regulate the activity of a number of inflammation-related molecules; therefore, SNPs affecting this gene could either directly or indirectly affect multiple inflammatory signaling pathways.

Taken together, these results showed that the -144 polymorphism altered HSP90beta expression levels and was associated with the cellular inflammatory response and the severity of organ dysfunction following injury. Unlike other susceptibility genes, HSP90beta could influence multiple inflammation-related genes simultaneously, thus showing more dramatic overall effects as a result of SNPs. Therefore, HSP90beta expression could be used to predict the severity of infections in patients following injury, and it could also be used to more efficiently diagnose and monitor high-risk patients.
